# PDK4 and nutrient responses explain muscle specific manifestation in mitochondrial disease

**DOI:** 10.1002/ctm2.70404

**Published:** 2025-07-18

**Authors:** Swagat Pradhan, Takayuki Mito, Nahid A Khan, Sofiia Olander, Aleksandra Zhaivoron, Thomas G McWilliams, Anu Suomalainen

**Affiliations:** ^1^ Stem Cells and Metabolism Research Program, Faculty of Medicine, University of Helsinki Helsinki Finland; ^2^ Department of Cellular Physiology Graduate School of Medical Sciences, Kyushu University Fukuoka Japan; ^3^ HiLife, University of Helsinki Helsinki Finland; ^4^ Department of Anatomy Faculty of Medicine, University of Helsinki Helsinki Finland; ^5^ HUS Diagnostic Centre, Helsinki University Hospital Helsinki Finland

**Keywords:** progressive external ophthalmoplegia, nutrient signaling, mitochondrial disease, integrated stress response, mitochondrial myopathy, pyruvate dehydrogenase kinase

## Abstract

**Background:**

Mitochondria elicit various metabolic stress responses, the roles of which in diseases are poorly understood. Here, we explore how different muscles of one individual—extraocular muscles (EOMs) and quadriceps femoris (QFs) muscles—respond to mitochondrial disease. The aim is to explain why EOMs atrophy early in the disease, unlike other muscles.

**Methods:**

We used a mouse model for mitochondrial myopathy (“deletor”), which manifests progressive respiratory chain deficiency and human disease hallmarks in itsmuscles. Analyses included histology, ultrastructure, bulk and single‐nuclear RNA‐sequencing, metabolomics, and mitochondrial turnover assessed through in vivo mitophagy using transgenic mito‐QC marker mice crossed to deletors.

**Results:**

In mitochondrial muscle disease, large QFs upregulate glucose uptake that drives anabolic glycolytic one‐carbon metabolism and mitochondrial integrated stress response. EOMs, however, react in an opposite manner, inhibiting glucose and pyruvate oxidation by activating PDK4, a pyruvate dehydrogenase kinase and inhibitor. Instead, EOMs upregulate acetyl‐CoA synthesis and fatty‐acid oxidation pathways, and accumulate lipids. In QFs, *Pdk4* transcription is not induced.‐ Amino acid levels are increased in QFs but are low in EOMs suggesting their catabolic use for energy metabolism. Mitophagy is stalled in both muscle types, in the most affected fibers.

**Conclusions:**

Our evidence indicates that different muscles respond differently to mitochondrial disease even in one individual. While large muscles switch to anabolic mode and glycolysis, EOMs actively inhibit glucose usage. They upregulate lipid oxidation pathway, a non‐optimal fuel choice in mitochondrial myopathy, leading to lipid accumulation and possibly increased reliance on amino acid oxidation. We propose that these consequences of non‐optimal nutrient responses lead to EOMatrophy and progressive external ophthalmoplegia in patients. Our evidence highlights the importance of PDK4 and aberrant nutrient signaling underlying muscle atrophies.

## Graphical abstract

Mitochondrial disease triggers opposite responses in different muscles. Mitochondrial integrated stress response (ISRmt) and aerobic glycolysis characterize large muscles. Eye muscles show no ISRmt but activate PDK4—inhibitor of glucose oxidation—thereby upregulating beta‐oxidation, which is non‐optimal in mitochondrial disease and can explain eye muscle atrophy in mitochondrial disease.

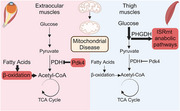

## 1 BACKGROUND

Mechanisms that cause diseases to manifest in a highly tissue‐specific manner are insufficiently understood. Especially the variability of manifestations is characteristic for mitochondrial diseases, which is surprising, as mitochondria are essential in all cell types.^1^ Specific metabolic requirements of different cell types and disease–signal‐dependent stress responses are arising as factors for tissue‐specificity.^2^

Skeletal muscle is commonly affected in adults with mitochondrial diseases. In these mitochondrial myopathies (MM), the extraocular muscles (EOMs) are typically the first to manifest weakness and progress to atrophy, leading to progressive external ophthalmoplegia and blepharoptosis.^1^ However, large skeletal muscles, such as quadriceps femoris (QFs) show exercise intolerance, but no atrophy.^3^ The underlying mechanisms of the especial sensitivity of EOMs to mitochondrial disease remains unknown. Autopsy studies in patients with MM have indicated replacement of the muscle fibers by fibrotic tissue. Also, disease‐relevant animal models have been lacking.

MM is typically caused by defects of mtDNA expression, such as defects of mitochondrial DNA (mtDNA): mutations in mitochondrial tRNAs or heteroplasmic single large‐scale mtDNA deletions.^1^ Also, nuclear‐encoded proteins of mtDNA maintenance cause MM. These include, for example, replicative helicase or polymerase of mtDNA (Twinkle, TWNK; DNA polymerase gamma, POLG) and different enzymes regulating mitochondrial nucleotide pools.^4^, ^5^, ^6^ All of these underlying defects lead to respiratory chain (RC) deficiency.^1^ However, other causes of RC deficiency, such as variants in RC enzyme subunits, most commonly cause a central nervous system disease, and do not typically affect EOMs. Therefore, mechanisms of MM and progressive external ophthalmoplegia link to responses elicited by mtDNA expression defects instead of general RC deficiency.

The “Deletor” mice, which express constitutively a dominant Twinkle patient mutation^7^ replicate well the morphological and molecular characteristics of adult‐onset MM, and results from these mice have provided preclinical data for pilot studies on patients.^8^ Data from Deletors indicate that large limb muscles exhibit a stage‐wise upregulation of mitochondrial integrated stress response, ISRmt^8^, ^9^, ^10^, ^11^, ^12^, ^13^, ^14^ that remodels metabolism in the whole cells. The early activation of ISRmt is marked by robust transcriptional upregulation of ATF transcription factor target genes encoding MTHFD2, an enzyme of mitochondrial folate cycle, as well as metabokines FGF21 and GDF15. The second stage is dependent on prior induction of FGF21 and induces synthesis of serine (PHGDH, PSAT1), nucleotides, the transsulfuration pathway (CTH, CBS), and modifies one‐carbon metabolism remarkably.^11^ mTORC1 (mechanistic target of rapamycin complex 1) is active in the most affected fibers that also show stalled mitochondrial turnover.^12^, ^15^ Until now, ISRmt has been expected to be generalized to all affected muscle types.

Here we used the Deletor mice to explore molecular mechanisms of progressive external ophthalmoplegia. We report that the small oxidative EOMs induce opposite responses from large, more glycolytic QFs, with especial difference in fuel choices. The data indicate high tissue‐specificity of mitochondrial stress responses even between different muscles.

## 2 METHODS

### 2.1 Mouse models

The transgenic Deletor mouse model was previously generated in the C57BL/6 congenic background.^7^ These mice express constitutively murine *Twnk* cDNA, carrying a dominant mutation (homologous to a MM patient mutation, leading to a 13‐amino acid duplication of amino acids p.353‐365 in the protein). The controls were wild‐type (WT) littermates of Deletor mice. All experiments were conducted using 22–24‐month‐old male mice derived from Twinkle‐D line.^7^

Transgenic *mito*‐QC mice were described in detail in McWilliams et al.^16^ They express *mito*‐QC, a fluorescent in vivo mitophagy reporter. We produced Deletor mice homozygous for *mito*‐QC transgene as in.^15^ Control mice were WTs expressing *mito*‐QC.

### 2.2 Respiratory chain enzyme activity analyses

Histochemical cytochrome c oxidase (COX) and succinate dehydrogenase (SDH) activity analyses were performed on frozen tissue sections (10 µm) from QFs and EOMs as in Forsström et al.^11^ QFs were incubated for 30 min for COX at room temperature (RT) and 40 min for SDH at 37°C, and EOMs for 20 min for COX at RT and 40 min for SDH at 37°C. After staining, the sections were dehydrated in ascending alcohols, xylene‐treated and mounted. Imaging was done by light microscopy (Axioplan 2 Universal Microscope, Zeiss).

### 2.3 Mitophagy immunostaining and assay

For mitophagy assay, EOMs and QFs were collected from 23 months old control and Deletor mice, homozygous for mito‐QC. The tissues were fixed in 3.7% paraformaldehyde at pH 7.0 in 0.2 M HEPES for 24 h and cryoprotected in 30% (w/v) sucrose in phosphate buffered saline at 4°C. The tissue samples were embedded in O.C.T. compound (SAKURA) and frozen in liquid nitrogen‐cooled isopentane. Following that, 8 µm thick frozen sections were blocked for 1 h with 5% BSA in 0.4% TritonX‐100/PBS and incubated with rat anti‐LAMP1 antibody (sc‐19992, Santa Cruz) or mouse anti‐p62 antibody (ab56416, Abcam) for 15 h, at 4°C. Tissue sections were then incubated with anti‐rat IgG Alexa fluor 633 (A‐21094, Thermo Fisher Scientific) or anti‐mouse IgG2a CF405S (SAB4600476, Sigma) for 1 h at RT. The tissue sections were nuclear counterstained with Hoechst 33342 (62249, Thermo Fisher Scientific) or DRAQ5 (ab108410, Abcam) and slides were mounted with Vectashield Antifade Mounting Medium (H‐1000, Vector). Confocal images were acquired using a laser‐scanning confocal microscope (LSM880, Zeiss). For the quantification of confocal images, each pixel of the confocal images was classified into mitochondria (GFP positive), red‐only puncta (GFP negative, mCherry positive and LAMP1 positive), lysosome (LAMP1 positive), p62 accumulation (p62 positive) or nuclei (Hoechst positive) by Ilastik 1.4.0 software^17^ and “ImageMath” and “IdentifyPrimaryObjects” modules of CellProfiler 4.2.5 software.^18^ Then, all the pixels were quantified by “MeasureObjectSizeShape” modules of CellProfiler 4.2.5 software.

### 2.4 Immunostaining and quantification

For phospho‐S6 immunohistochemistry staining, 8–9 µm frozen muscle sections were stained as in Forsström et al.^11^ except that the phospho‐S6 antibody (2215, Cell Signaling Technology) was used with dilution 1:250 in place of the antibody specified in the original study and imaging was done by light microscopy (Axioplan 2 Universal Microscope, Zeiss).

For MTCO1/SDHA/pPDHA1/MTHFD2 staining, tissues were fixed in 10% buffered formalin and embedded in paraffin. Antigen retrieval was done using Tris‐EDTA pH 9.0 for MTCO1/SDHA/pPDHA1 staining and 10 mM sodium citrate, 0.05% Tween 20 pH 6.0 for MTHFD2 staining. The samples were blocked with 3% BSA and 5% normal goat serum in 0.1% Tween‐20/TBS for 1 h and subsequently blocked with M.O.M (mouse on mouse) reagent in 0.1% Tween‐20/TBS for 1 h at RT. The sections were incubated overnight with 1:100 dilutions of MTCO1 (ab14705, abcam), SDHA (ab14715, abcam), 1:250 dilution of Phospho‐Ser293‐PDHA1 (ab177461, abcam) or 1:300 dilution of MTHFD2 (ab151447, abcam) at 4°C. Tissue sections were washed with 0.1% Tween‐20/TBS and incubated with 1:200 dilution of anti‐mouse IgG1 biotin (ab97238, abcam), anti‐mouse IgG2a (#A21136, Invitrogen) and anti‐rabbit IgG (#A11011, Invitrogen) for 2 h at 4°C. The tissue sections were then incubated with 1:100 dilution of Streptavidin (#S21374, Thermo Fisher Scientific) for 2 h at 4°C. Slides were mounted with Vectashield Antifade Mounting Medium (H‐1000, Vector). Images were taken in Zeiss Axioimager with apotome.

pPDH quantifications were done using “MeasureImageIntensity” module of CellProfiler 4.2.5. pPDH pixels from COX‐/SDH+ fibers were excluded by manually cropping them using ImageJ software and analysing them through CellProfiler 4.2.5 software.

Fiber size quantification was done using COX/SDH stained images. Outline of the fibers were selected manually using “Freehand selection” tool in ImageJ. After selection, the fiber area was measured using “Measure” tool in ImageJ.

### 2.5 Electron Microscopy

For electron microscopy, fresh QFs and EOMs samples were processed as in Ignatenko et al.^19^ Briefly, 1–2 mm^3^ of QFs and eyeball with EOMs were dissected out and fixed overnight at 4°C with 2.5% glutaraldehyde (G6257, Sigma) in 0.1 M sodium phosphate buffer. After washing, samples were post‐fixed with 1% non‐reduced osmium tetroxide, dehydrated in ethanol and embedded in epoxy resin. The area of interest for the ultrastructural analyses were selected by analyzing the sections with light microscope. Ultrathin (60–90 nm) sections were cut on grids and stained with uranyl acetate and lead citrate. TEM micrographs were acquired with JEOL 1400 transmission electron microscopy running at 80 kV, with a bottom‐mounted CCD camera (Orius SC 1000B, AMETEK‐Gatan Inc.), with images of 4008 × 2672 pixels.

### 2.6 mtDNA copy number and deletions

To isolate total cellular DNA from frozen QFs and EOMs samples, a standard proteinase K and phenol‐chloroform extraction method was used. mtDNA copy number was quantified by quantitative PCR (qPCR) by amplification of mtDNA fragment MTRNR1 against nuclear‐DNA‐encoded RBM15. 25 ng of DNA was used per PCR reaction, and all qPCR assays were run in triplicates. The load of mitochondrial DNA (mtDNA) deletions was determined using primers (Table S3) and PCR conditions as in Khan et al.^12^ Briefly, mtDNA deletion load was determined by long‐range and short‐range PCR. For long range PCR, amplification of partially deleted molecules was analysed by primers chosen from non‐deleted mtDNA region between MTRNR2 and MTND1 genes and amplifying 15.78 kb of the mtDNA. Short range PCR was done to determine total mtDNA amount using amplification of a 582‐bp fragment from the non‐deleted region of mtDNA.

### 2.7 RNA isolation and RNAseq analysis

Total RNA from QFs and EOMs samples was extracted from snap frozen tissues in Trizol reagent (Invitrogen) and homogenized with Precellys Lysing kit—Tissue homogenizing CKMix (Bertin Technologies) and Precellys w‐24 (Bertin Technologies). All the EOM muscles from each mouse were dissected identically as described in.^20^ Briefly, EOMs were dissected after opening the skull and removing the bony material alongwith harderian gland under Olympus stereomicroscope. The different EOM muscles from each mouse (recti, obliques, retractor bulbi and levator palpebrae) were combined in an eppendorf tube and snap frozen in liquid nitrogen prior to RNA extraction. The RNA extracted was treated with DNAse and purified through RNeasy Qiagen minicolumns. The manufacturer's instructions were followed for the extraction. The RNA quality was analysed using TapeStation. Samples with a minimum of RIN value 7 were used for RNA‐sequencing.

Bulk‐RNA sequencing of QFs and EOMs samples was performed by 3′ UTR RNA “Bulkseq” sequencing as in Kang et al.^21^ Differential gene expression analysis was done by Deseq2 package in R environment^22^ using default settings. A negative binomial linear model and Wald test were used to produce *p*‐values. Low‐expression outliers were removed using Cook's distance to optimize the *p*‐value adjustment and finally, multiple testing adjustment of *p*‐values was done with the Benjamini–Hochberg procedure.

For cis‐regulatory analysis, cytoscape^23^ (version 3.10.1) with plug‐in iRegulon^24^ was used. For motif enrichment, genomic regions of 10 kb around the respective transcriptional start site were analysed and predicted transcription factors were filtered with a normalized enrichment score > 3.0.

For single nuclear‐RNA sequencing (snRNAseq), nuclei were extracted from QFs as in Santos et al.^25^ modified as follows. Nuclear dye 7‐amino‐actinomycin D (00‐6993‐50, eBioscience) was used to stain the nuclei prior to sorting with Sony SH800Z Cell Sorter. The quality and integrity of the nuclei was assessed with brightfield microscopy prior to the sequencing. Single cell gene expression profiles were studied using 10× Genomics Chromium Single Cell 3′RNAseq platform. The Chromium Single Cell 3′RNAseq run and library preparation were performed using the Chromium Next GEM Single Cell 3′ Gene Expression version 3.1 Dual Index chemistry. The Sample libraries were sequenced on Illumina NovaSeq 6000 system using read lengths: 28 bp (Read 1), 10 bp (i7 Index), 10 bp (i5 Index) and 90 bp (Read 2).

Data processing and analysis were performed using 10x Genomics Cell Ranger v7.1.0^26^ pipelines. Of the Cell Ranger pipelines, we used the “cellranger mkfastq” to produce FASTQ (raw sequence data) files and “cellranger count” to perform alignment, filtering and UMI counting. mkfastq was run using the Illumina bcl2fastq v2.2.0 and alignment was done against the mouse genome mm10. The cellranger aggr pipeline was used to aggregate individual samples into a single feature‐barcode matrix. Data analysis was performed using Scanpy (v1.8.2).^27^ Initial quality control included filtering out nuclei with fewer than 200 gene transcripts, genes expressed in fewer than 3 nuclei, and nuclei with high mtDNA transcript content. Data were normalized for sequencing depth and then log‐transformed. Dimensionality reduction was conducted using Uniform Manifold Approximation and Projection (UMAP)^28^ for visualization. Clustering was performed with the Leiden algorithm.^29^ Nuclei were assigned to clusters by comparing identified marker genes with known cell type‐specific markers described by Petrany et al.^30^ Differential expression analysis identified marker genes for each cluster using the Wilcoxon rank‐sum test, with adjustments for multiple testing via the Benjamini–Hochberg method. This analysis was carried out within the Scanpy framework.

### 2.8 Targeted metabolomics analysis

Snap frozen tissues of QFs and EOMs from WT/Deletor mice were used to extract metabolites for targeted metabolomics. The samples were homogenized using 2 mL Precellys homogenization tube (Bertin Technologies) with 2.8 mm ceramic (zirconium oxide) beads and 500 µL of cold extraction solvent (acetonitrile:methanol:Milli‐Q Water; 40:40:20). Subsequently, samples were homogenized using a tissue homogenizer (Bertin Technologies) for three cycles (30 s at 5500 rpm with 60 s pause at 4°C), followed by centrifugation at 14000 rpm at 4°C for 5 min. The supernatant was loaded into a Phenomenex, Phree Phospholipid removal 96 ‐well plate (Part No. 8E‐S133‐TGB) and passed through using a robotic vacuum. The filtrate was transferred into polypropylene tubes and placed into a nitrogen gas evaporator to completely dry the solvent. Dried samples were suspended with 40 µL of extraction solvent, vortexed for 2 min and transferred into high‐performance liquid chromatography glass‐autosampler vials. Finally, 2 µL of the sample was injected into a Thermo Vanquish UHPLC system coupled with a Q‐Exactive Orbitrap quadrupole mass spectrometer equipped with a heated electrospray ionization (H‐ESI) source probe from Thermo Fischer Scientific. For chromatographic separation, a SeQuant ZIC‐pHILIC column (2.1 × 100 mm, 5 µm polymer; Merck) was used. Gradient elution was performed with a flow rate of 0.100 mL/min using 20 mM ammonium hydrogen carbonate adjusted to pH 9.4 with ammonium solution (25%) as mobile phase A and acetonitrile as mobile phase B. The gradient elution began with 20% mobile phase A and 80% mobile phase B and was maintained until 2 min. Then, mobile phase A was gradually increased from 20% to 80% until 17 min, followed by a decrease from 80% to 20% in mobile phase A from 17.1 min and maintained up to 24 min. The column oven and auto‐sampler temperatures were set to 40 ± 3°C and 5 ± 3°C, respectively.

Mass spectrometry (MS) was used with a heated electrospray ionization (H‐ESI) source using polarity switching and the following settings: resolution of 70 000 spray voltages of 4250 V for positive mode and 3250 V for negative mode, sheath gas flow rate of 25 arbitrary units (AU), auxiliary gas flow rate of 15 AU, sweep gas flow rate of 0, capillary temperature of 275°C, and S‐lens RF level of 50.0. The instrument was controlled with the Xcalibur 4.1.31.9 software (Thermo Fischer Scientific).

In data processing, peak integration was performed using TraceFinder 5.1 software (Thermo Fischer Scientific), and the confirmed retention times of 462 metabolites in an in‐house library developed using the library kit MSMLS‐1EA (Merck) were used. The peak area data were exported as an excel file for further analysis. Data quality was monitored throughout the run using a pooled sample as quality control (QC), which was prepared by pooling 5 µL from each suspended sample and interspersed throughout the run as every 10th sample. After integration of QC data with TraceFinder 5.1, detected metabolites were checked for peak, and % RSD were calculated. An acceptance limit was set at ≤20%.

Blank samples for carryover were injected after every fifth randomized sample to monitor the metabolites' carryover effect and were calculated against the mean QC area. The acceptance limit was set at ≤20% for each metabolite. Background % noise was calculated with respect to the first blank against the mean QC area, and the acceptance limit was set at ≤20% for each metabolite.

Further, the data were explored and analysed using Metaboanalyst^31^ site following the recommended settings. Metabolites with missing values in ≥20% of the samples were excluded from further analysis. Normalization by sum of sample peaks was used to normalize the data and log transformation (glog2) and autoscaling (mean‐centred and divided by standard deviation of each variable) was used.

Pathway enrichment analysis of metabolomics dataset was done for all increased metabolites and all decreased metabolites regardless of significance values using recommended settings of pathway analysis module in Metaboanalyst.

### 2.9 Quantification and statistical analysis

Data are represented as median with interquartile range. Analyses were performed with unpaired two tailed student's *t*‐test. Data normality was assessed using the Shapiro–Wilk test. Homogeneity of variances was evaluated with the *F*‐test. If the data did not meet the assumption of normality, the non‐parametric Mann–Whitney *U* test was used to calculate the *p*‐value. A *p*‐value < 0.05 was considered statistically significant.

All statistical analysis except for bulk and single‐nuclear RNAseq and metabolomics were calculated using Prism 9.3.0 software. The analyses of bulk RNAseq were performed with R and snRNAseq by Python. Metabolomics analyses were done using metaboanalyst; graphs were made with R, Python and Graphpad Prism version 9.3.0. Heatmaps were generated using pHeatmap package in R, Z‐scores calculated from scaled reads per base; box plots with ggplot2 package and Graphpad Prism; volcano plots using “EnhancedVolcano” and ggplot2 function in R.

## 3 RESULTS

### 3.1 Hallmarks of mitochondrial myopathy in EOMs

**FIGURE 1:**
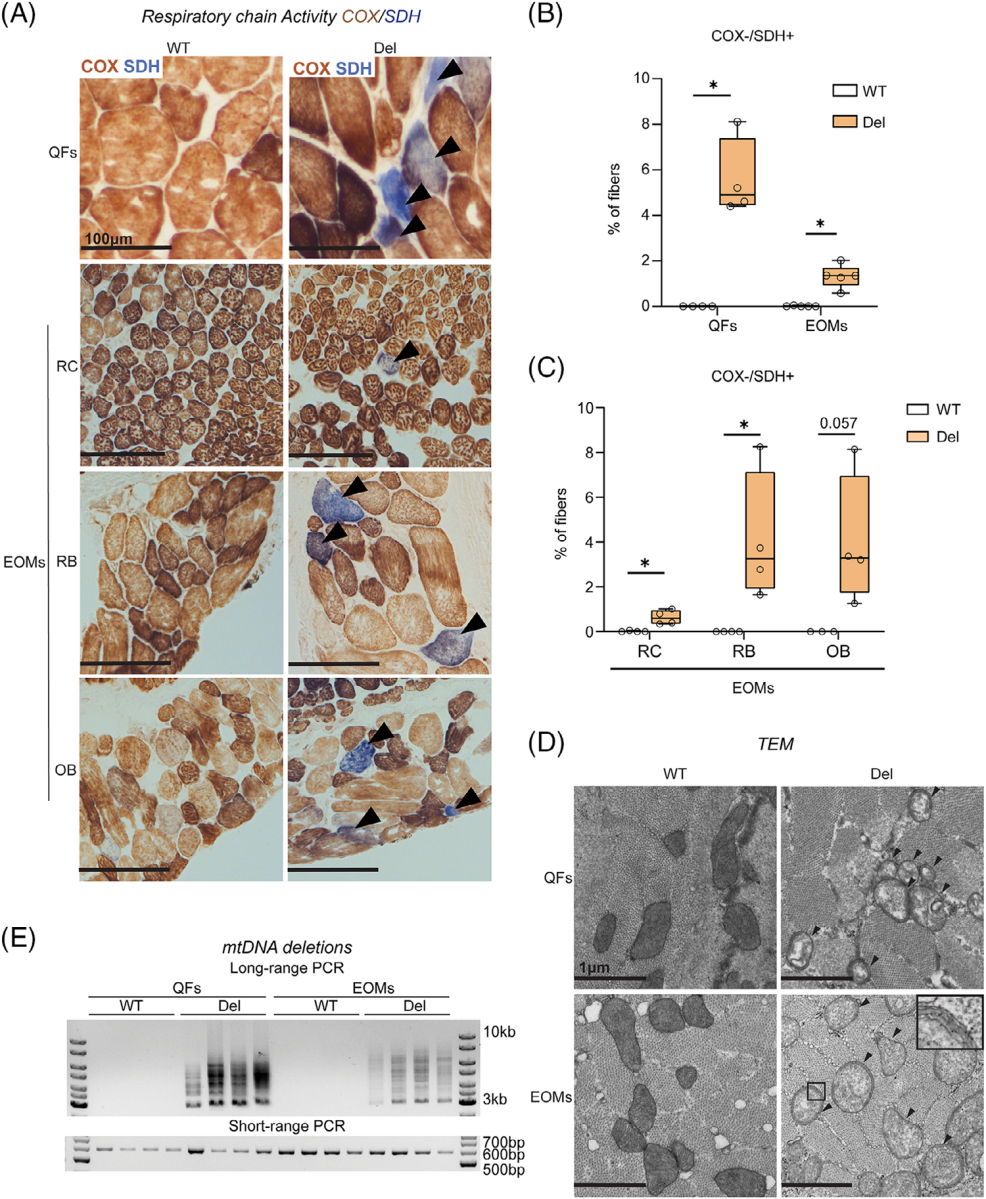
Hallmarks of mitochondrial myopathy in extraocular muscles. (A) Histochemical analysis of cytochrome c oxidase (COX; brown) and succinate dehydrogenase (SDH; blue) activities of quadriceps femoris (QFs) and extraocular muscles (rectus (RC) and retractor bulbi (RB) muscle and oblique (OB) muscle). Arrowheads indicate COX‐negative and SDH‐positive muscle fibers. Scale bars: 100 µm. (B) Amount of COX‐negative and SDH‐positive muscle fibers in QFs and EOMs (≥ 500 fibers counted per mouse in QFs; ≥500 in EOMs; *n* = 4 (QFs), *n* = 5 (EOMs)). (C) Amount of COX negative and SDH positive muscle fibers in EOMs (≥684 fibers for rectus (RC) muscle and 168 fibers for retractor bulbi (RB) of EOMs were counted per mouse and 90 fibers of oblique (OB) muscle of EOMs were counted per mouse; *n*≥3). (D) Mitochondrial ultrastructure. Electron micrograph of Deletor and WT QFs and EOMs. Arrowheads point to abnormal mitochondria. Inset: high magnification showing onion‐ring like structures. Scale bars: 1 µm. Multiple mtDNA deletions. Long‐range PCR (15.7 kbp) of mtDNA in Deletor QFs and EOMs. Short‐range PCR of mtDNA showing non‐deleted D‐loop region (582 bp). Data information: In (B–C), data are presented as boxplots. Whiskers represent minimum and maximum values, and horizontal line represents median. *p ≤ 0.05, **p ≤ 0.01, ***p ≤ 0.001, ****p ≤ 0.0001 (Student's *t*‐test).

Deficiency of RC complex IV (cytochrome c oxidase, COX; partially encoded by mtDNA) is a histological hallmark of MM, often accompanied by increased activity of nuclear‐encoded complex II, succinate dehydrogenase (SDH). The histochemical activity analysis on frozen muscle sections showed that the EOMs of Deletors showed 1% and QFs of the thigh 5% of COX‐/SDH+ fibers (Figure 1A,B). COX deficiency and increase of SDH amount were also evident at the protein level (immunofluorescence analysis; MTCO1 antibody for COX; SDHA for SDH) in both EOMs and QFs (Figure S1A). Mouse EOMs comprise of eight muscles (four rectus, two oblique, levator palpebrae and retractor bulbi). The different EOMs showed variable amounts of RC deficient fibers with the retractor bulbi and oblique muscles having ∼3% and rectus muscles (∼0.5%) (Figure 1C). Electron microscopic analysis revealed abnormal cristae, reduced crista density and multiple membranes in mitochondria of QFs (Figure 1D), whereas the mitochondria in EOMs had multilayered onion ring‐like membranes with abnormal / absent cristae (Figure 1D). In some Deletor EOMs fibers, the mitochondria exhibited tubular cristae with vacuolization (Figure S1B).

Long‐PCR amplification of mtDNA showed multiple products, indicative of multiple‐sized mtDNA deletions in both EOMs and QFs (Figure 1E). mtDNA copy number did not show a significant change in either muscle type (Figure S1C). Fiber size analysis did not show significant differences in fiber sizes in EOMs at this stage. However, on rare occasions, COX‐/SDH+ fibers showed atrophic features in Deletor EOMs (Figure S1D,E).

Collectively, these results indicate that both EOMs and QFs exhibit typical signs of MM in Deletor mice. The Deletor EOMs did not show widespread muscle fiber atrophy at this stage of MM, suggesting them to be a valuable model to elucidate early responses to MM.

### 3.2 Stalled autophagy/mitophagy in EOMs with mitochondrial dysfunction

**FIGURE 2:**
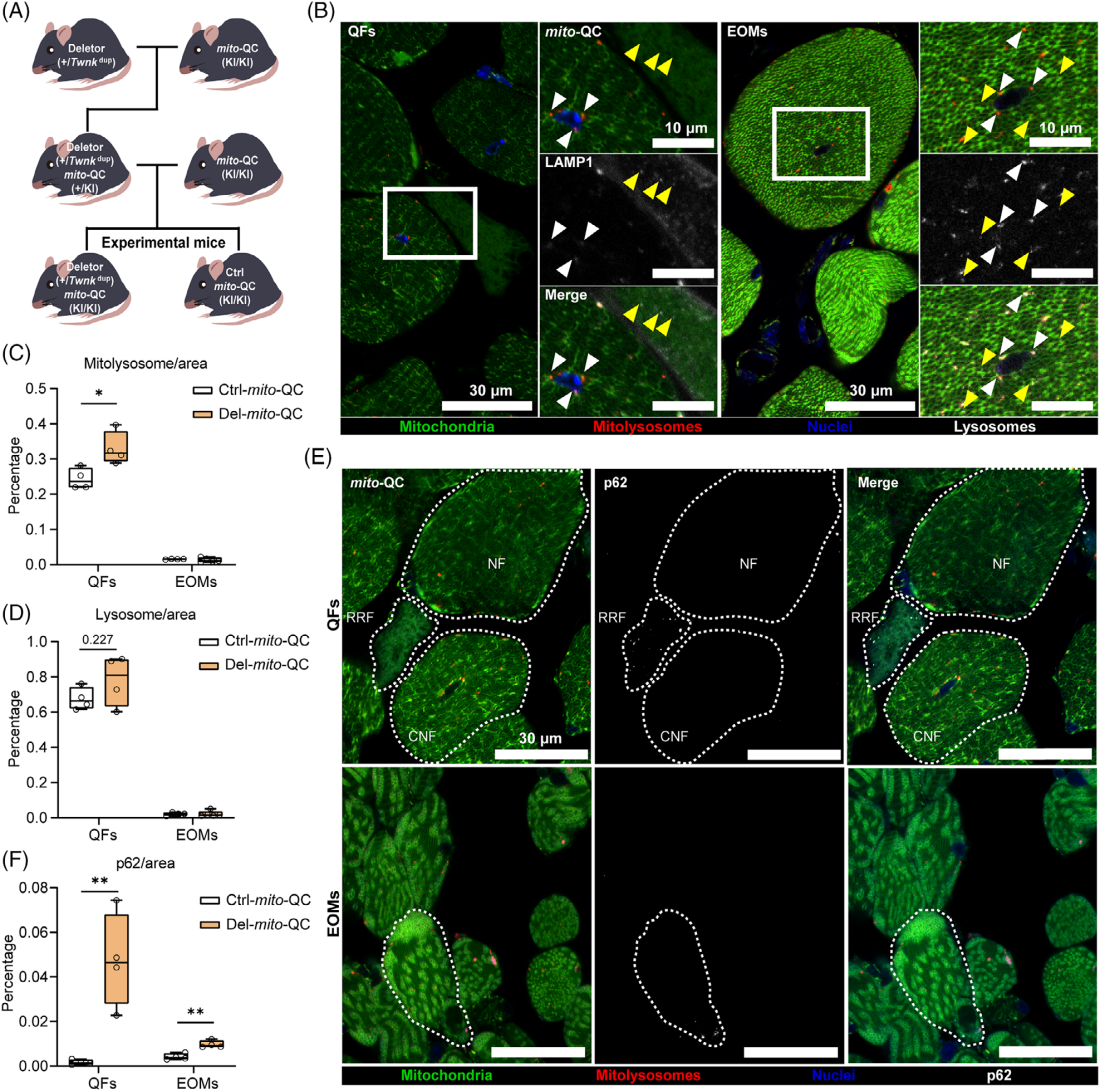
Mitophagy in quadriceps femoris and extraocular muscles in vivo. (A) Design of Deletor mice expressing *mito‐*QC reporter gene. Mice carrying a homologous mutation to MM patients (in‐frame duplication of amino acids 353–365 in Twinkle helicase) crossed with transgenic mice expressing mitophagy reporter mCherry‐GFP‐FIS1^101‐152^ (*mito*‐QC), expressed from *Rosa26* locus^16^ to generate Deletors with heterozygous *mito*‐QC gene. Obtained male mice were crossed to female homozygous *mito*‐QC mice to generate Deletor and control (Ctrl) mice with homozygous *mito*‐QC gene. (B) Mitophagy in quadriceps femoris (QFs) and extraocular muscles (EOMs) of Deletor *mito*‐QC mice. Representative confocal images of *mito*‐QC signals with high‐magnification enlargements (boxed with white lines) show mitochondrial network (green) and mitolysosomes (red). Mitolysosomes (white arrowheads) are confirmed to be colocalized with lysosomal marker protein lysosome‐associated membrane protein 1 (LAMP1) by immunostaining. Ragged‐red fibers show mitophagy stalling and accumulation of lysosomes (yellow arrowheads). Central nuclear fibers (CNFs) show elevated mitophagy around the centralized nuclei in QFs. Scale bars: 30 µm (lower magnification) and 10 µm (higher magnification). (C) Quantification of the total area of mitolysosomes in QFs and EOMs, normalized by muscle fiber area. (D) Quantification of the total area of lysosomes in QFs and EOMs, normalized by muscle fiber area. (E) Autophagic flux. Representative images of p62 immunostaining showing RRF‐specific p62 aggregation in QFs and the sporadic aggregation in EOMs of Deletor mice with homozygous *mito*‐QC. Fiber borders for central nuclear fiber (CNF), normal fiber (NF) and ragged‐red fiber (RRF) are indicated as dashed lines. Scale bars: 30 µm. (F) Quantification of area of p62 aggregates in QFs and EOMs, normalized by muscle fiber area. Deletor mice with homozygous *mito*‐QC (Del‐*mito*‐QC) and their control littermates (Ctrl‐*mito*‐QC); twenty regions of interest from each mouse, four different mice per genotype were quantified. Average values of each mouse. Data information: In (C–D, F), data are presented as boxplots. Whiskers represent minimum and maximum values, and horizontal line represents median. *p ≤ 0.05, **p ≤ 0.01, ***p ≤ 0.001, ****p ≤ 0.0001) (Student's *t*‐test).

Mosaic dysfunction of mitophagy is an important hallmark of MM, both in patient skeletal muscles and Deletor QFs.^15^ In the centrally nucleated fibers (CNFs), mitophagy is active and increased, while in the most affected “ragged‐red” fibers (RRFs), mitophagy is stalled. No changes in mitophagy are present in the structurally normal fibers in the same muscle tissue.^15^ To assess mitophagy in EOMs, we crossed Deletor mice and their controls with transgenic mice expressing constitutively *mito*‐QC, an in vivo mitophagy reporter.^16^ The ubiquitous expression of *mito*‐QC successfully illuminated both mitochondrial structures and mitolysosomes in EOMs and QFs of 23‐month‐old mice (Figure 2A,B). Although ragged‐red fibers (RRFs) were rare in Deletor EOMs, when present, they exhibited stalled mitophagy; by contrast, increased mitophagy was consistently observed in CNFs (Figure 2B; Figure S2A,S2B), similar to QFs (Figure 2B; Figure 4S2C). However, the total level of mitophagy was not elevated in EOMs (Figure 2C). In the Deletor QFs, the elevated overall mitophagy reflected the high amount of CNFs compared to RRFs (Figure 2B,C). The total level of lysosomes in Deletor QFs did not show a significant change but a small increasing trend, whereas no change was observed in Deletor EOMs (Figure 2D). CNFs showed increase in number of lysosomes per fiber (Figure S2B,S2C) while RRFs exhibited increased lysosomal levels when normalized to fiber area in both QFs and EOMs (Figure S2D).

The p62, an autophagy receptor that accumulates upon decreased autophagic flux, showed aggregation and increased total level in Deletor QFs (Figure 2E,F;Figure S2E), supporting decreased autophagic/mitophagic flux in RRFs. In Deletor EOMs, p62 total amount showed a small yet statistically significant increase (Figure 2F), even though p62 aggregation typically found in RRFs in QFs was absent. Instead, we found sporadic aggregation in morphologically normal fibers and COX negative SDH positive fibers in Deletor EOMs (Figure 2F;Figure S2E).

These results indicate that aberrant mitophagy does not explain the vulnerability of EOM to atrophy.

### 3.3 Extraocular muscles have a distinct transcriptomic response to mitochondrial disease

**FIGURE 3:**
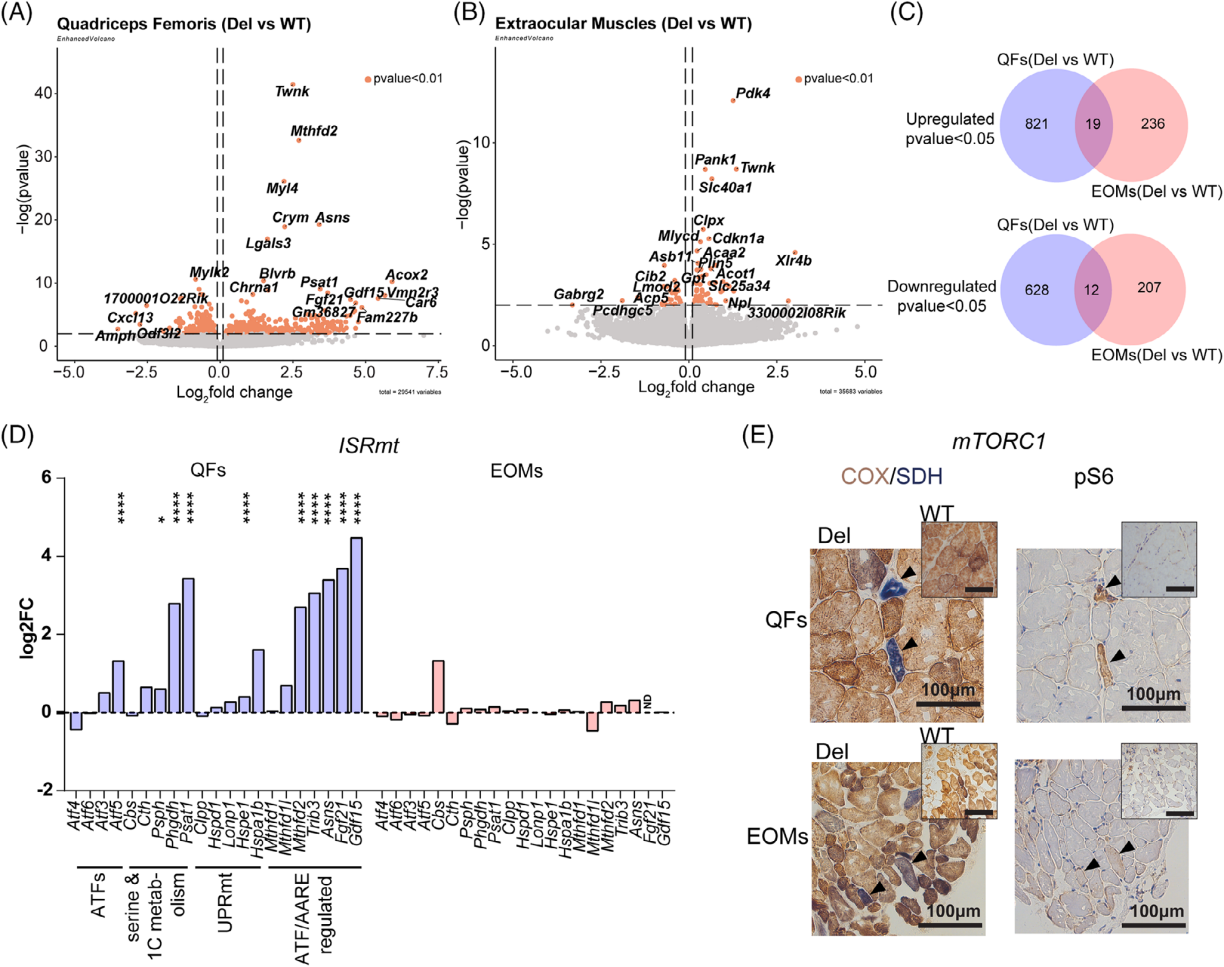
Extraocular muscles (EOMs) with MM have a distinct transcriptomic response to MM than quadriceps femoris (QFs) muscles and lack mitochondrial integrated stress response (ISRmt). (A) Volcano plot depicting the fold‐change and statistical significance of the transcriptomic changes between WT and Deletor groups in quadriceps femoris (QFs). The dotted line marks the significance threshold at P < 0.01. (B) Volcano plot depicting the fold‐change and statistical significance of the transcriptomic changes between WT and Deletor groups in extraocular muscle (EOMs). The dotted line marks the significance threshold at *p* < 0.01. (C) Venn diagram; significantly co‐upregulated and co‐downregulated transcripts between QFs (Del vs. WT) and EOMs (Del vs. WT). (D) Mitochondrial integrated stress response in QFs and EOMs of Deletors (gene list is curated based on Forsström et al.^11^). *Fgf21* was not detected (ND) in EOMs dataset. (E) mTORC1 activity measured by S6‐phosphorylation in EOMs and QFs of WT and Deletors. Scale bars: 100 µm.

To elucidate the tissue‐specific molecular pathomechanisms in MM, we performed RNAseq analysis of QFs and EOMs samples. Our analysis of Deletor EOMs showed that 255 transcripts were upregulated and 219 downregulated (*p* < 0.05) (Table S1). In the Deletor QFs, transcription of 840 genes was upregulated and 640 downregulated (*p* < 0.05) (Table S1). However, when we compared the two muscle types, they shared only 19 of the upregulated genes and 12 of the downregulated ones (Figure 3C). 44 genes were significantly changed in opposite directions in these two groups (Table S1), among them were the metabolic regulator *Pdk4* and iron exporter *Slc40a1*.

### 3.4 ISRmt genes are not induced in EOMs

The transcriptome profile revealed that ISRmt was not induced in EOMs (Figure 3D; Figure S3A), while it was activated in QFs. These include activating transcription factor 5 (*Atf5*), de novo serine biosynthesis (*Psat1, Phgdh, Psph*), mitochondrial chaperone (*Hspe1*), ATF‐regulated asparagine synthetase (*Asns*), mitochondrial folate cycle (*Mthfd2*), tribbles pseudokinase 3 (*Trib3*), fibroblast growth factor 21 (*Fgf21*) and growth differentiation factor 15 (*Gdf15*). MTHFD2 was induced mosaically in COX‐/SDH+ fibers of Deletor EOMs and QFs (Figure S3B). Notably, in Deletor EOMs, MTHFD2 induction was absent in many COX‐negative/SDH‐positive fibers, supporting a lack of ISRmt activation in these fibers. Oxidative stress response genes and proteases did not show significant changes in Deletor EOMs with the exception of induction of *Clpx* protease, which was downregulated in QFs, all pointing to a distinct response (Figure S3C).

Additionally, we examined mTORC1 activation by immunostaining its downstream target phosphorylated S6 (pS6) and COX/SDH staining in the serial sections. QFs show strong induction of pS6 in COX‐negative and SDH‐positive fibers, while in EOMs the phosphorylation is not prominent (Figure 3E), suggesting that ISRmt is activated in a muscle type‐specific manner in MM. The potential protective role of ISRmt for QFs would therefore not exist in EOMs.

### 3.5 PDK4 and lipid oxidation activated in EOMs of MM mice

**FIGURE 4:**
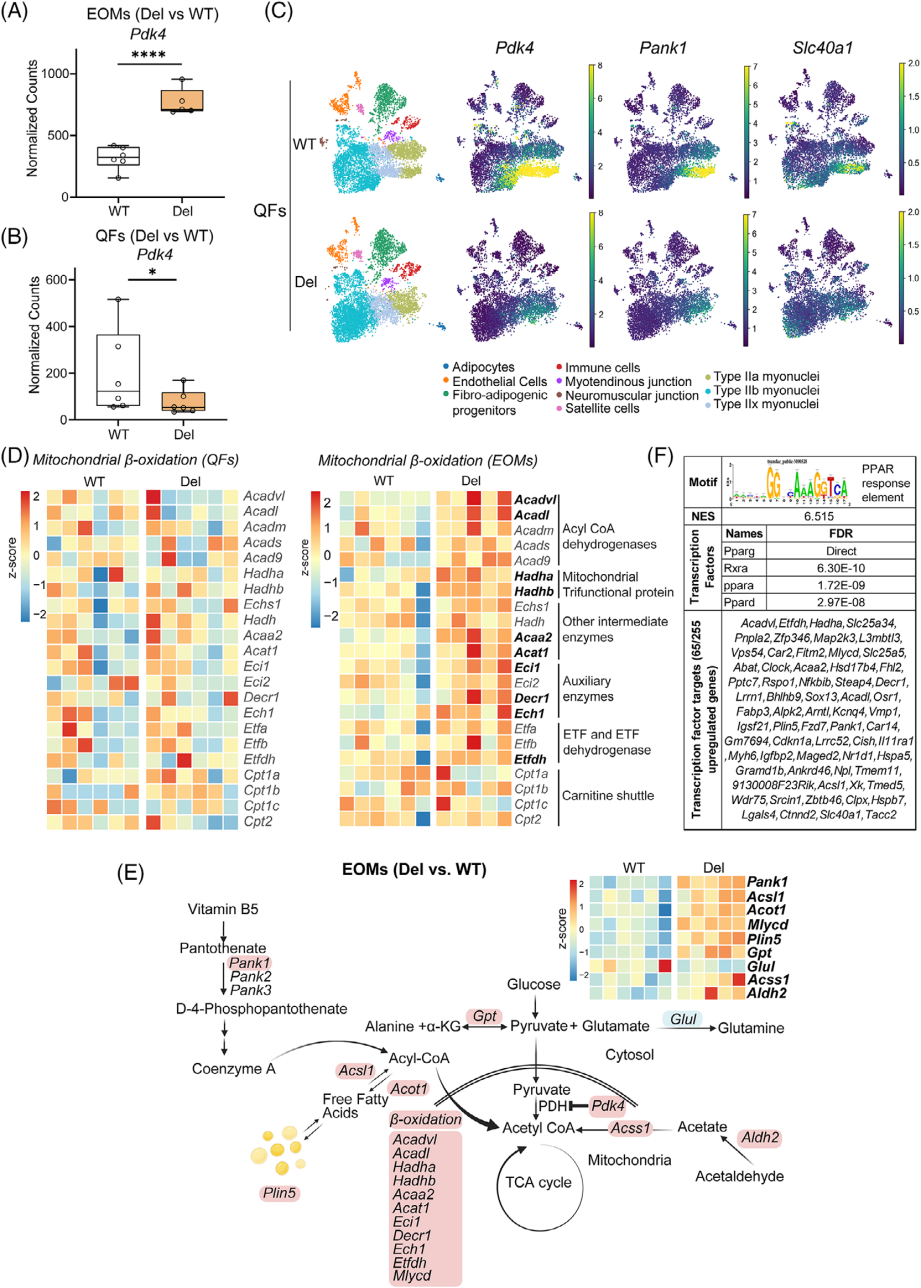
Pdk4 and lipid oxidation genes are induced in Deletor extraocular muscles (EOMs). (A) *Pdk4* transcripts; normalized counts from RNAseq data in EOMs (Del vs. WT). (B) *Pdk4* transcripts; normalized counts from RNAseq data in QFs (Del vs. WT). (C) Heatmap showing transcript expression levels of *Pdk4*, *Pank1* and *Slc40a1* in different nuclei populations in QFs of WT and Del. (D) Heatmap depicting mitochondrial beta‐oxidation changes in QFs and EOMs (Del vs. WT). Highlighted transcripts are significantly changed (*p* < 0.05). (E) Transcript changes related to pyruvate metabolism in EOMs. Transcripts highlighted are significantly changed (*p* < 0.05) (Red; upregulated, Blue; downregulated). Heatmap panel shows the significantly changed transcripts. Illustrations made with BioRender.com. (F) Motif enrichment analysis of upregulated genes in Deletor EOMs showing enrichment of PPAR family transcription factors and Rxrα target motif in 65/255 upregulated genes (NES: Normalized enrichment score). Data information: In (A, B), data are presented as boxplots. Whiskers represent minimum and maximum values and horizontal line represents median. *p ≤ 0.05, **p ≤ 0.01, ***p ≤ 0.001, ****p ≤ 0.0001) (Wald test).

*Pdk4* was the most significantly upregulated transcript in Deletor EOMs dataset when compared to their WT littermates (Figure 4A). However, in the large QFs, *Pdk4*‐mRNA was downregulated, both in bulk and single‐nuclear RNA‐sequencing, in all fiber types, especially in the oxidative type IIa fibers (Figure 4B,C). As PDK4 is an active inhibitor of pyruvate dehydrogenase (PDH) activity, acting as a glucose and fatty acid metabolic regulator,^32^ we analysed the transcript levels of glycolytic and beta‐oxidation enzymes. Indeed, mitochondrial beta‐oxidation genes were widely upregulated in the Deletor EOMs, but not in QFs (Figure 4D). These included acyl‐CoA dehydrogenases (*Acadvl, Acadl*), mitochondrial trifunctional protein (*Hadha, Hadhb*), mitochondrial ketoacyl CoA‐thiolase (*Acaa2*), acetoacetyl‐CoA thiolase (*Acat1*) and other auxiliary enzymes (*Eci1, Decr1, Ech1*) (Figure 4D,E;Figure S4A). Expression levels of glycolytic enzymes preceding pyruvate oxidation were unchanged in Deletor EOMs and QFs. Enzymes connected to the glucose‐alanine cycle (*Gpt, Glul*) showed aberrant gene expression (Figure 4E) pointing towards anomalous pyruvate metabolism.

Acyl‐CoA, pyruvate and acetyl‐CoA metabolic pathways were specifically affected in Deletor EOMs. The long‐chain fatty‐acid‐coenzyme A ligase (*Acsl1*), acyl‐CoA hydrolase (*Acot1*) and acyl‐CoA catabolic enzyme transcripts (*Mlycd, Pcca*) were induced, suggesting Acyl‐CoA imbalance (Figure 4E). Perilipin 5 (*Plin5*), a lipid droplet coating protein shown to recruit mitochondria to lipid droplets,^33^ was also upregulated (Figure 4E). Coenzyme A synthesis regulatory enzyme (*Pank1*) and acetaldehyde to acetyl CoA synthesis pathway enzymes (*Aldh2, Acss1*) were induced in Deletor EOMs (Figure 4E). Further, a cis‐regulatory analysis of upregulated genes in EOMs predicted enrichment of PPAR response elements (PPREs) motif, a PPAR/RXR heterodimer binding target in 65 of 255 upregulated genes in the EOM dataset (Figure 4F and Table S1). These changes along with activation of *Pdk4* and beta‐oxidation genes suggest decreased pyruvate to acetyl‐CoA conversion and activation of alternate pathways to maintain acetyl‐CoA levels and a shift to catabolic metabolism and lipid oxidation.

Phosphorylation of PDK4 target PDH (pPDH) at a primary inhibitory serine site (293) was increased in COX‐/SDH+ positive fibers and also showed an overall increased trend even in non COX‐/SDH+ muscle fibers (Figure S4B,S4C). pPDH was mosaically increased in QFs, even if *Pdk4* transcript was downregulated in both bulk and single nuclear‐ RNAseq of the muscle; some COX‐/SDH+ fibers had high pPDH, while non‐COX‐/SDH+ fibers showed an overall decrease in PDH phosphorylation (Figure S4D,S4E).

Iron exporter ferroportin (*Slc40a1*) expression was robustly upregulated in Deletor EOMs, but downregulated in QFs, suggestive for disease‐modified iron homeostasis in EOMs (Figure 4C). The iron levels were too low to be quantitatively analysed by histological means in EOMs (not shown). In QFs snRNAseq, both *Slc40a1* and *Pank1* transcription were downregulated, especially in type IIa oxidative fibers (Figure 4C).

Taken together, our data indicate that the mtDNA maintenance defect in EOMs leads to an activation of pathways that promote substrate utilization from glucose to fatty acids and upregulate pathways that generate acetyl‐CoA and beta‐oxidation. In EOMs this effect is likely mediated by activation of PDK4.

### 3.6 Fatty acid accumulation in the EOMs

Next, we studied if the transcriptomic changes were reflected in metabolite levels. For this, we detected and analysed a targeted metabolome of ∼ 230 metabolites in both QFs and EOMs of Deletors (Table S2).

**FIGURE 5:**
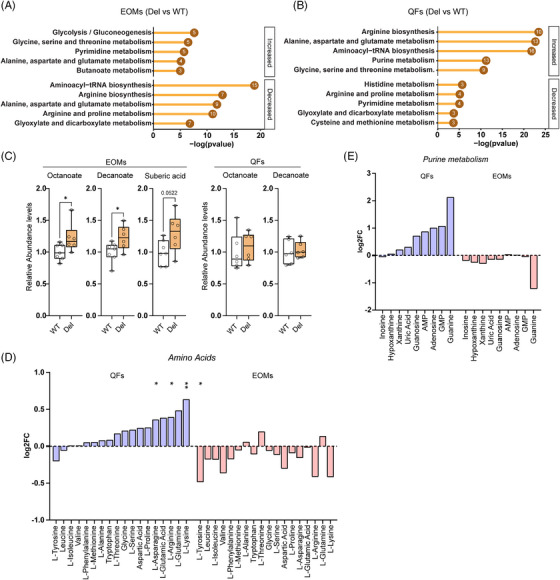
Metabolomic profiles are different in Deletor extraocular muscles (EOMs) and quadriceps femoris (QFs). (A) Pathway enrichment analysis of all the changed metabolites between WT and Deletors in EOMs. Numbers in the circles denote the number of metabolites changed in the pathway; *n* = 6 (WT), 7 (Del). (B) Pathway enrichment analysis of all the changed metabolites between WT and Deletors in QFs. Numbers in the circles denote the number of metabolites changed in the pathway; *n* = 6. (C) Fatty acid relative abundance levels in EOMs and QFs. (D) Comparison of amino acid levels between QFs and EOMs of Deletors. (E) Comparison of detected metabolites involved in purine metabolism between QFs and EOMs of Deletors. Data information: In (C–E), *p*‐value from *t*‐test of Metabolomics dataset calculated by metaboanalyst; *p ≤ 0.05, **p ≤ 0.01, ***p ≤ 0.001, ****p ≤ 0.0001 (Student's *t*‐test).

Pathway enrichment analysis showed a general increase in glycolysis intermediates in EOMs (Figure 5A;Figure S5A) consistent with the inhibition of pyruvate dehydrogenase by PDK4. Although not significant, pyruvate showed an increased trend in Deletor EOMs but decreased trend in QFs. Lactate did not show a change (Figure S5B). Also, significantly elevated fatty acid anions (octanoate and decanoate) in the EOMs suggest impaired fatty acid oxidation in MM (Figure 5C).

In QFs, pathway enrichment analysis revealed an overall increase in steady‐state amino acids levels and predicted increase of amino‐acyl tRNA synthetase pathway (Figure 5B,D). However, in EOMs, a general decreasing trend or no change in amino acids was present and their tRNA synthetase pathways were decreased (Figure 5D). Purine intermediates trended upwards in QFs, while EOMs showed either no change or decrease (Figure 5E).

These results indicate remarkably opposing metabolic rewiring in EOMs and QFs, glycolytic and anabolic in QFs and catabolic in EOMs.

## 4 DISCUSSION

Here, we report the different molecular metabolic signatures and nutrient choices of two different muscle types in response to mitochondrial myopathy. In patients, the first manifestation of the disease is progressive external ophthalmoplegia developing to atrophy of EOMs,^1^ which they consider as a severe symptom: the lack of eye movements and progressive blepharoptosis disturb not only vision but also social interactions. The mechanisms that explain the special sensitivity of EOMs to mtDNA expression defects have been unclear, mainly because the small muscles undergo atrophy during the disease and molecular or metabolic studies of fibrotic autopsy samples are mechanistically uninformative. The Deletor mice with dominant, homologous Twinkle mutation to that of MM‐patients ^4^, ^7^ develop a typical mitochondrial myopathy ^34^, ^35^ with accumulation of mtDNA deletions, presentation of respiratory chain deficient fibers and mitochondria with ultrastructural abnormalities in both large muscles and extraocular muscles. Previously, responses in Deletors were replicated in human patients, even serving as preclinical evidence for treatment trial in patients with the same homologous mutation.^8^ Importantly, the phenotypes observed in Deletor mice mimic the early‐stage disease in patients. In the case of EOMs, the Deletors do not show muscle atrophy and fibrosis that develop in the patients early on. Therefore, Deletor EOMs may offer a unique opportunity to study initial molecular and metabolic responses that underlie progressive external ophthalmoplegia in patients. We show that the molecular responses of EOMs and the thigh muscles are partially opposite, especially for nutrient metabolism. The large quadriceps femoris muscles are characterized by anabolic metabolism with glucose dependence: glucose uptake, glycolytic energy metabolism and upregulated glucose‐driven one‐carbon metabolism. The EOMs, however, show a catabolic signature: PDK4‐mediated inhibition of PDH and glucose metabolism, upregulated beta‐oxidation but insufficient capacity to use lipids for energy metabolism, leading to lipid accumulation. The lack of glucose or fatty acids as a fuel source challenge EOMs energy metabolism, increasing dependence on amino acid breakdown. Our evidence suggests that these catabolic responses and inability to shift to glucose‐driven stress responses lead to EOM atrophy and external ophthalmoplegia.

In the skeletal muscle, the regulation of pyruvate dehydrogenase complex activity plays an important role in glucose homeostasis and fuel selection.^36^ *Pdk4* transcript is the most significantly upregulated mRNA in EOMs which encodes PDK4, a key kinase that inhibits PDH. PDK4 activation is known to suppress carbon flux from glycolysis to the TCA cycle and activate beta‐oxidation as an alternate carbon source.^37^ PPAR‐family transcription factors regulate PDK4 as well as lipid and glucose metabolism^38^, ^39^ and indeed PPAR‐family transcription factors and RXRα are predicted to be the most significant upstream regulators of the transcriptome in EOMs. The next most significantly induced transcript, *Pank1*, codes for the enzyme that is the rate‐limiting step of coenzyme‐A synthesis, required for fatty acid metabolism. PANK1 is part of a p53‐regulated pathway, which downregulates glucose uptake via inhibiting glucose transporters.^40^, ^41^ Further, the third‐most significant change, ferroportin induction, suggests that in EOMs, mitochondrial myopathy induces a signal to export iron from the muscle. Recent data suggest that iron and glucose metabolism are co‐regulated by STAT3‐linked pathway,^42^ which has been reported in tumours to downregulate glucose oxidation via its direct target PDK4.^43^ These top pathways that come together in EOM transcriptome lead to inhibition of glucose oxidation and potential iron deficiency in EOMs, but are not induced in the QFs. The loss of metabolic flexibility as a consequence of PDK4 activation in EOMs has also been reported in diseases with severe secondary muscle loss, such as cancer cachexia and amyotrophic lateral sclerosis.^44^, ^45^ Our data on EOMs together with current literature emphasize the importance of muscle stress responses, nutrient metabolism and PDK4 as a contributor to muscle atrophy in general.

PDK4 mediated robust PDH phosphorylation (pPDH) in Deletor EOMs in fibers showing respiratory chain deficiency/loss of COX protein. In thigh muscles, however, PDH phosphorylation was inhibited in some respiratory chain deficient fibers. As our single nuclear RNASeq indicated decrease of *Pdk4* transcript in oxidative type‐II fibers, where it typically is expressed, a possibility remains that another kinase phosphorylates PDH in large muscles in mitochondrial myopathy. The variability of pPDH between different fiberss in QFs indicates that mitochondrial disease induces different responses in different muscle types and even in a mosaic manner in adjacent fibers. This was evident also for mitophagy response in the current and a previous study,^15^ highlighting the need of cell‐specific approaches when analysing metabolic responses of muscle.

PDK4 activation is known to be induced by starvation,^46^ suggesting an intriguing possibility that oxidative muscle fibers interpret deficient mtDNA expression as a lack of nutrients, and therefore upregulates fatty acids as their preferred fuel. The response in QFs supports this conclusion: fasting‐related metabokine FGF21 is secreted to blood circulation as part of their ATF5‐driven ISRmt response to mitochondrial disease.^11^, ^14^ This response is lacking from EOMs. FGF21 secretion has a systemic effect: it releases fatty acids from their storage tissues, adipose tissue and liver,^14^ increasing fatty acid availability for tissues. EOMs upregulate lipid oxidation pathway but have respiratory chain deficiency, which prevents fatty acid oxidation and results in consequent accumulation of lipids with lipotoxic potential.^47^ Lipid accumulation is a hallmark of mitochondrial myopathy in patients^48^, ^49^ and, as we report here, occur also in Deletor EOMs, making a lipotoxic mechanism and the lack of protective ISRmt as plausible contributos to the progression of ophthalmoplegia and EOMs atrophy.

Our results point to specific treatment strategies to slow down EOMs progression to atrophy. PDK4 inhibition, PDH activation or PPAR inhibition should likely be local, as systemic effects of such a key regulator of metabolism are deleterious. Indeed, pan‐PPAR activator bezafibrate has been found beneficial for large muscles of mice with mitochondrial myopathy^50^ but whether EOMs would respond similarly, or whether PPAR‐activation could actually increase progression of ophthalmoplegia, remains to be clarified. Dichloroacetic acid (DCA) is a PDK pan‐inhibitor, which alleviated lactic acidosis in mitochondrial myopathies, encephalopathies, and congenital lactic acidosis.^51^, ^52^  However, DCA trials on mitochondrial disease patients were halted due to an adverse side‐effect,^53^ development of peripheral neuropathy. The shared regulation of glucose and iron metabolic changes via STAT3, potentially also occurring in the EOMs, suggests preclinical testing of STAT3 inhibitors, currently being developed for cancer therapy.^54^

## 5 CONCLUSIONS

Our study reveals remarkable muscle‐ and fiber‐type specific responses to mitochondrial disease that direct opposite fuel oxidation preference in EOMs and thigh muscles. We propose that lack of energy metabolic flexibility, non‐optimal fuel choices, toxicity of accumulating lipids, together with muscle type‐specific inability to upregulate metabolic restorative stress response ISRmt explain the sensitivity of EOMs to atrophy. The data emphasize the complexity of mitochondrial disease mechanisms even in one tissue type, such as muscle. Further, our results highlight the potential importance of nutrient metabolism also as a potential contributor to other types of muscle atrophy involving PDH inhibition, such as cachexia or amyotrophic lateral sclerosis.

## AUTHOR CONTRIBUTIONS

*Conception and design of the study*: Swagat Pradhan, Nahid A Khan and Anu Suomalainen. *Acquisition and analysis of the data*: Swagat Pradhan, Takayuki Mito, Sofiia Olander, Aleksandra Zhaivoron and Thomas G McWilliams. *Drafting a significant portion of the manuscript or figures*: Swagat Pradhan, Takayuki Mito and Anu Suomalainen.

## CONFLICT OF INTEREST STATEMENT

The authors declare no conflicts of interest.

## ETHICAL APPROVAL

Animal Experimental Board of Finland, adhering to the directives set by the European Union, approved the animal experimentation (ESAVI/3686/2021).

## Supporting information



Figure S1. Mitochondrial myopathy in EOMs and QFs
Figure S2. Ragged‐red fibers (RRFs) in EOMs
Figure S3. Stress responses in QFs and EOMs
Figure S4. PDH phosphorylation in QFs and EOMs
Figure S5. Pyruvate and fatty acid metabolism are affected in EOMs with MM
Table S1. Transcriptome dataset with cis‐regulatory analysis (Attached separately)
Table S2. Metabolome dataset (Attached separately)
Table S3. Primers used in the study
Table S4. Reagents and resources used in the study

Supporting Information

Supporting Information

## Data Availability

The datasets have been deposited to the following databases. Bulk‐RNA‐seq datasets: European nucleotide archive (ENA) with accession no. PRJEB73353. Metabolomics datasets: Metabolomics workbench^55^ with study ID ST003145 (https://doi.org/10.21228/M8WF0F). All key reagents used in this study are listed in the Table S4.
