# Supplementary material for: PDK4 and nutrient responses explain muscle specific manifestation in mitochondrial disease
Source: Clin Transl Med. 2025 Jul 18;15(7):e70404. doi: 10.1002/ctm2.70404 (PMC12274114; doi:10.1002/ctm2.70404)
Supplement: Supplementary file 1 — Figure S1. Mitochondrial myopathy in EOMs and QFs Figure S2. Ragged‐red fibers (RRFs) in EOMs Figure S3. Stress responses in QFs and EOMs Figure S4. PDH phosphorylation in QFs and EOMs Figure S5. Pyruvate and fatty acid metabolism are affected in EOMs with MM Table S1. Transcriptome dataset with cis‐regulatory analysis (Attached separately) Table S2. Metabolome dataset (Attached separately) Table S3. Primers used in the study Table S4. Reagents and resources used in the study [file CTM2-15-e70404-s001.docx]

**PDK4 and nutrient responses explain**

**muscle specific manifestation in mitochondrial disease**

Swagat Pradhan^1^, Takayuki Mito^1,5^, Nahid A Khan^1^, Sofiia Olander^1^, Aleksandra Zhaivoron^1^, Thomas G McWilliams^1,2,4^, Anu Suomalainen^1,2,3^

^1^Stem Cells and Metabolism Research Program, Faculty of Medicine, University of Helsinki, 00290 Helsinki, Finland

^2^HiLife, University of Helsinki, 00290 Helsinki, Finland

^3^HUS Diagnostic Centre, Helsinki University Hospital, 00290 Helsinki, Finland

^4^Department of Anatomy, Faculty of Medicine, University of Helsinki, 00290 Helsinki, Finland

^5^Department of Cellular Physiology, Graduate School of Medical Sciences, Kyushu University, 812-8582 Fukuoka, Japan.

**SUPPLEMENTARY MATERIAL**

**CONTENTS**

**Figure S1. Mitochondrial myopathy in EOMs and QFs**

**Figure S2. Ragged-red fibers (RRFs) in EOMs**

**Figure S3. Stress responses in QFs and EOMs**

**Figure S4. PDH phosphorylation in QFs and EOMs**

**Figure S5. Pyruvate and fatty acid metabolism are affected in EOMs with MM**

**Table S1. Transcriptome dataset with cis-regulatory analysis (Attached separately)**

**Table S2. Metabolome dataset (Attached separately)**

**Table S3. Primers used in the study**

**Table S4. Reagents and resources used in the study**

**
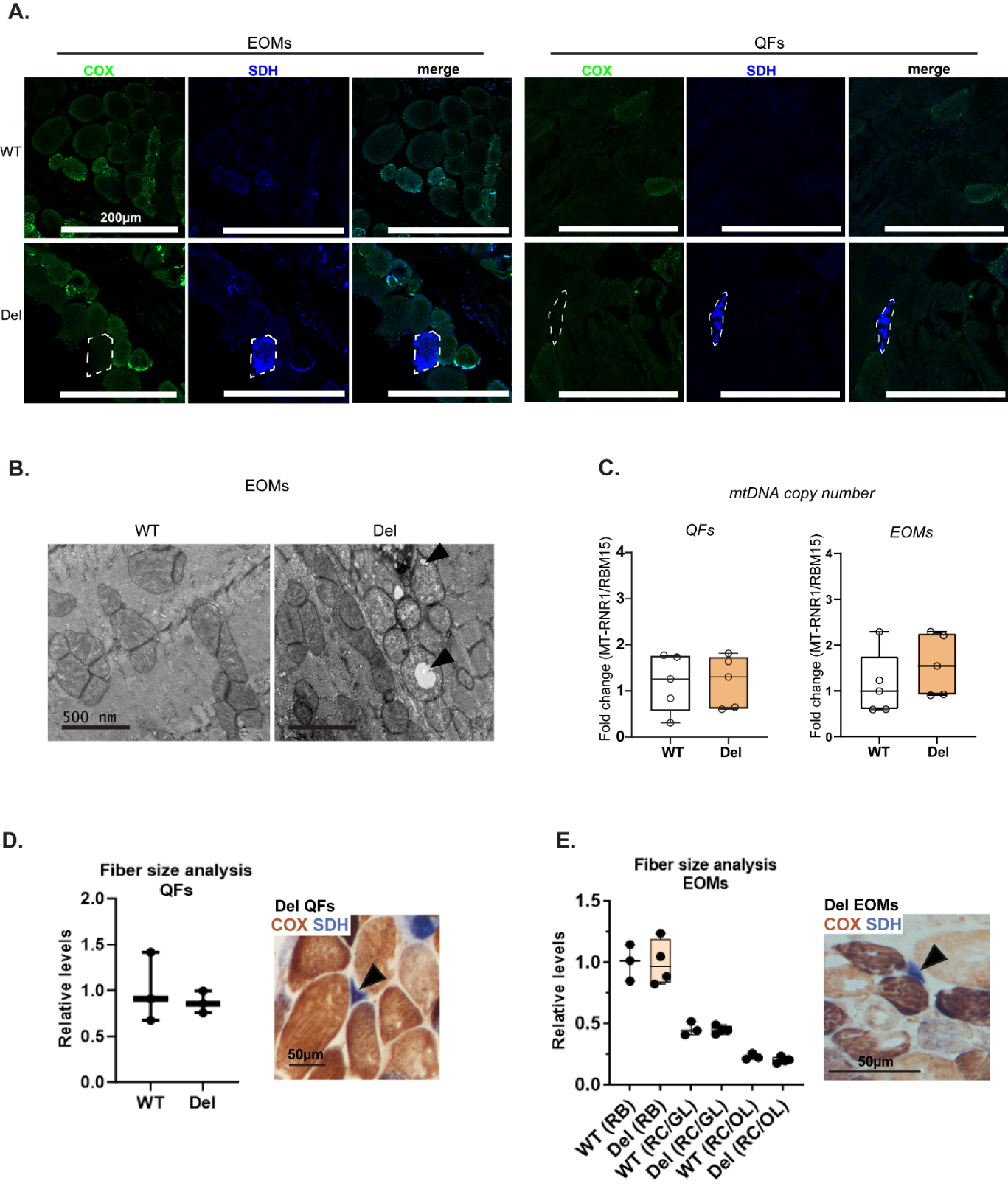
**

**Figure S1. Mitochondrial myopathy in EOMs and QFs**

1. Immunostaining of mitochondrial complex IV protein; MTCOI (green) and Complex II protein; SDHA (blue) in QFs and EOMs. Highlighted fibers show MTCO1 negative and SDHA positive fibers. Scale bars:200 µm
2. Mitochondrial ultrastructure. Electron micrograph of WT and Deletor EOMs. Arrows showing abnormal mitochondria with vacuolisation in Deletor EOMs. Scale bars:100 nm
3. mtDNA copy number in QFs and EOMs of WT and Deletor mice (n=5)
4. Fiber size analysis of QFs (n=3) (≥ 300 fibers counted per mouse). COX/SDH image represents fiber with atrophic feature.
5. Fiber size analysis of EOMs (n=3 (WT), 4 (Del)) (≥ 100 fiber analyzed for retractor bulbi (RB)and Rectus/global layer (RC/GL), ≥ 50 fibers for Rectus/Orbital layer (RC/OL) per mouse). COX/SDH image represents rare fiber with atrophic feature

**
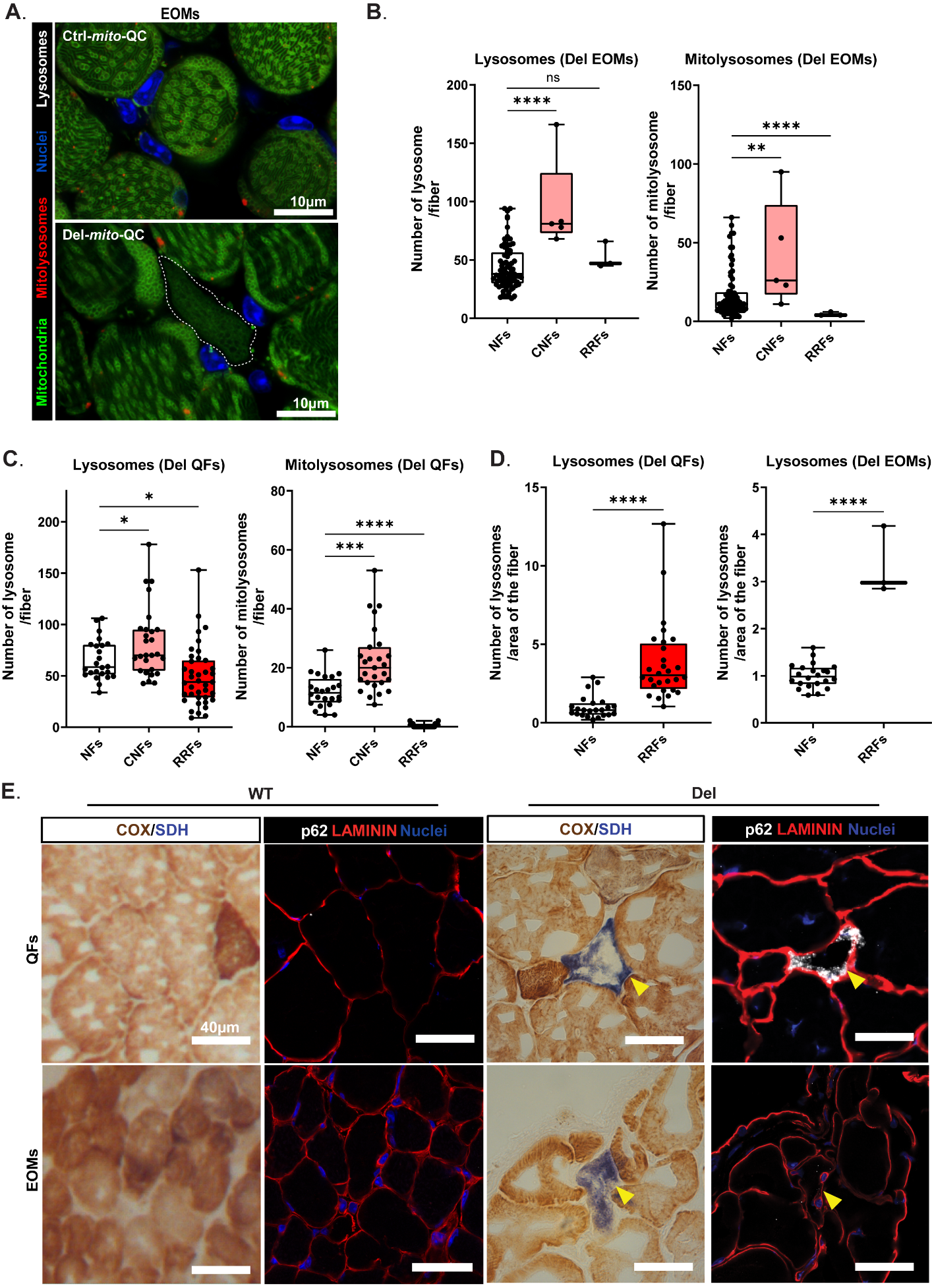
**

**Figure S2. Ragged-red fibers (RRFs) in EOMs**

1. Mitophagy in RRFs. Representative confocal images of *mito*-QC signals showing stalled mitophagy in RRF in EOMs
2. Lysosome and mitolysosome amount quantification of normal fibers (NFs), central nuclei fibers (CNFs) and ragged red fibers (RRFs) in EOMs.
3. Lysosome and mitolysosome amount quantification of normal fibers (NFs), central nuclei fibers (CNFs) and ragged red fibers (RRFs) in QFs.
4. Lysosome amount quantification of normal fibers (NFs) and ragged red fibers (RRFs) normalized to fiber area in QFs and EOMs.
5. Autophagy flux. Representative image showing accumulation of p62 in COX negative and SDH positive by immunostaining in QFs and EOMs. Scale bars:40 µm

Data information: In (B, C and D), data are presented as bar plots. Whiskers represent standard deviation and height of the bar represents mean. *P ≤ 0.05, **P ≤ 0.01, ***P ≤ 0.001, ****P ≤ 0.0001) (Student’s t-test)

**
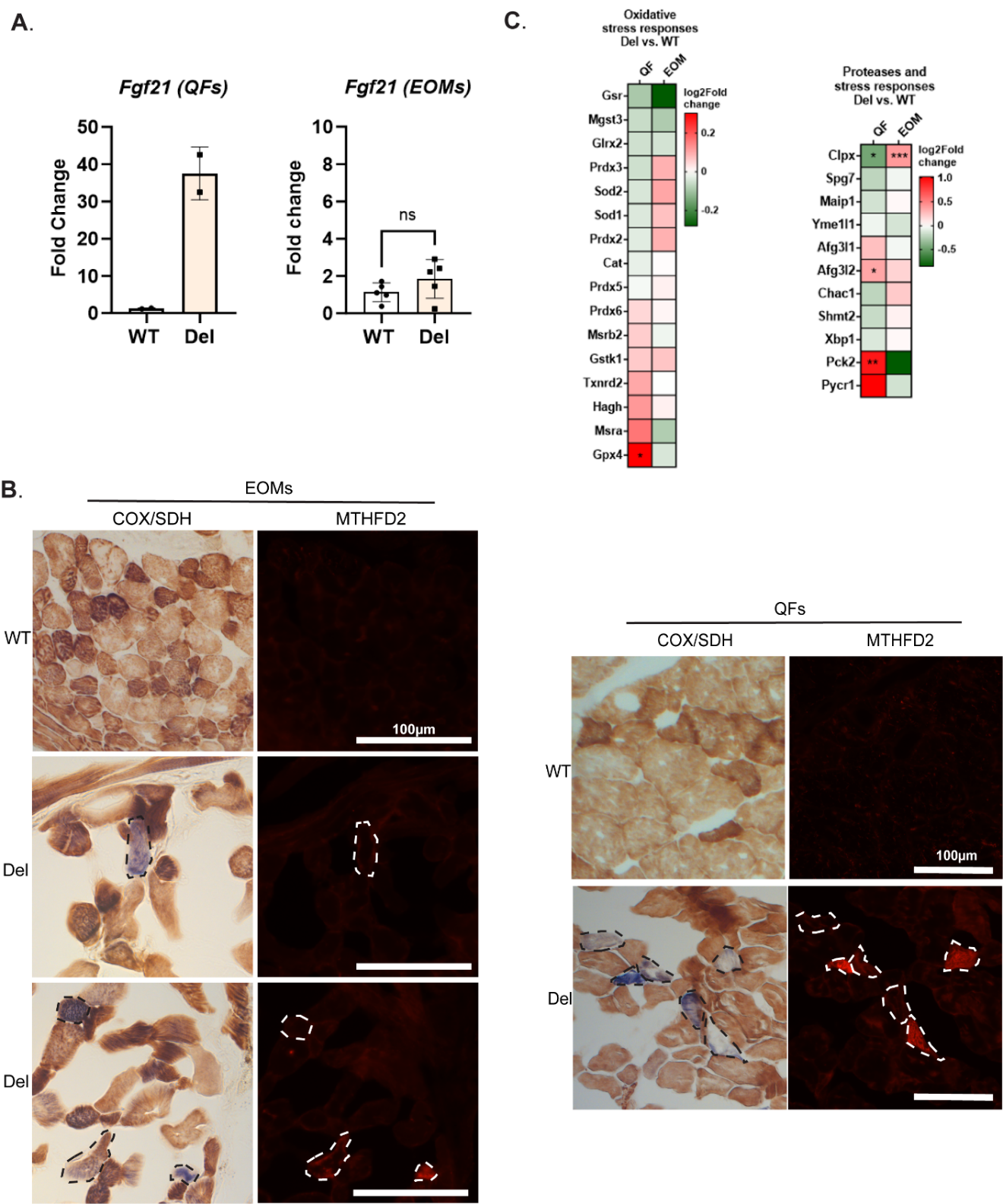
**

**Figure S3. Stress responses in QFs and EOMs**

1. *Fgf21* gene expression analysis by qPCR relative to beta-actin in QFs and EOMs. (Student’s t-test)
2. MTHFD2 immunostaining in QFs and EOMs of WT and Deletors. Scale bars: 100 µm
3. Oxidative stress response markers and proteases transcripts expression heatmap. Heatmap boxes with star * mark are significant. *P ≤ 0.05, **P ≤ 0.01, ***P ≤ 0.001, ****P ≤ 0.0001) (Wald test)

**
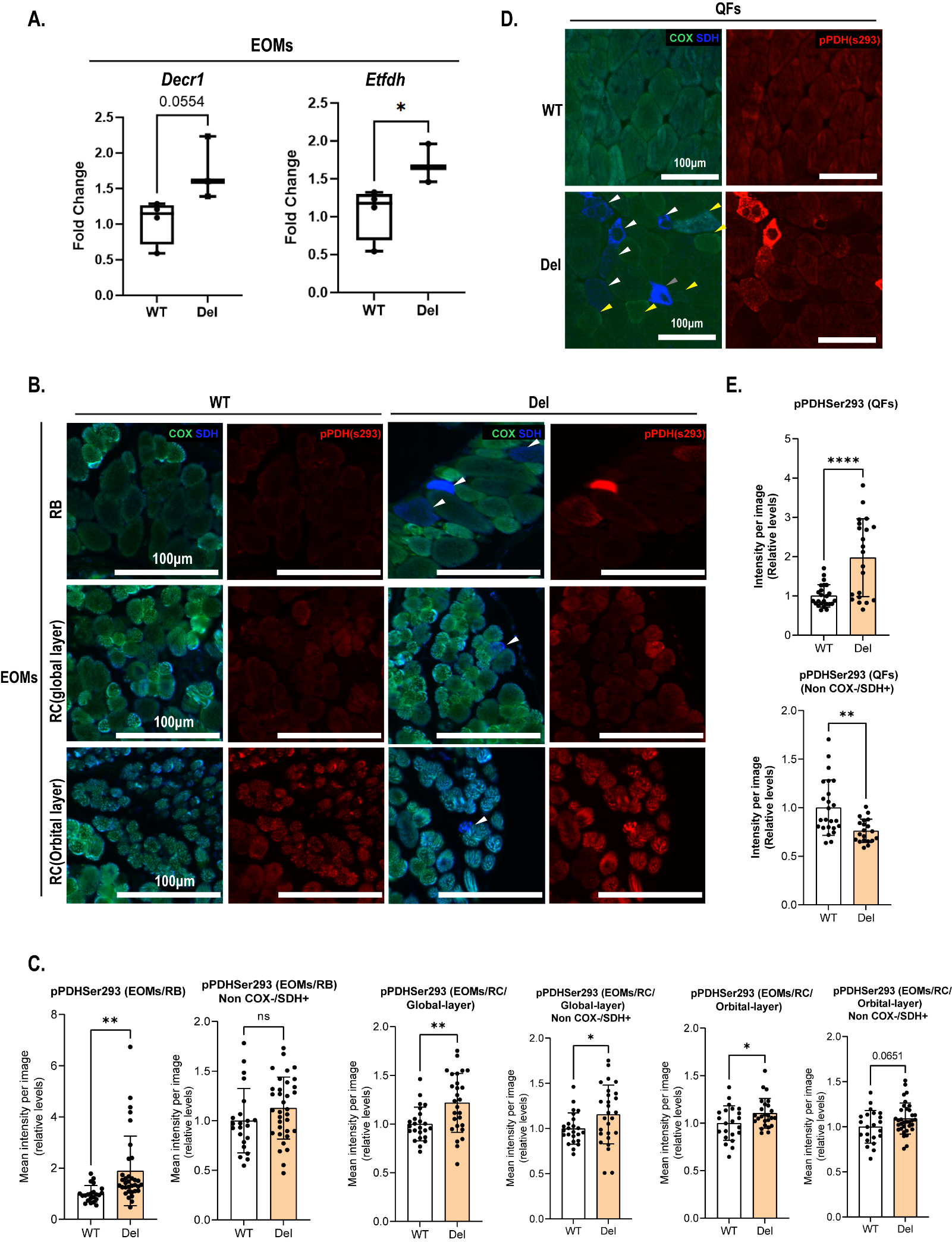
**

**Figure S4. PDH phosphorylation in QFs and EOMs**

1. Gene expression analysis using qPCR of beta-oxidation genes (*Decr1, Etfdh*) relative to beta-actin from EOMs (n≥3)
2. Immunofluorescence analysis (MTCO1 for COX, SDHA for SDH; PDH phosphorylation (Ser293) antibodies) in different muscle regions (RB: Retractor Bulbi, RC: Rectus) of EOMs of WT and Deletors. (White arrows: COX-/SDH+ and phospho-PDH positive fibers). Scale bars:100 µm.
3. Quantification of PDH phosphorylation (Ser293) intensity in all fibers (left) and in non-COX-/SDH+ fibers (right) (n≥3).
4. PDH phosphorylation, a PDK4 target. Immunofluorescence analysis (MTCO1 for COX, SDHA for SDH; PDH phosphorylation (Ser293) antibodies) in QFs showing mosaic changes in Deletor QFs. (White arrows: COX-/SDH+ and phospho-PDH positive fibers, Gray arrows: COX-/SDH+ and phospho-PDH negative fibers, Yellow arrows; phospho-PDH negative fibers). Scale bars:100 µm
5. Quantification of PDH phosphorylation (Ser293) intensity in all fibers and in non-COX-/SDH+ fibers (n≥3).

Data information: In (A, C, E), data are presented as bar plots. Whiskers represent standard deviation and height of the bar represents mean. *P ≤ 0.05, **P ≤ 0.01, ***P ≤ 0.001, ****P ≤ 0.0001) (Student’s t-test)

**
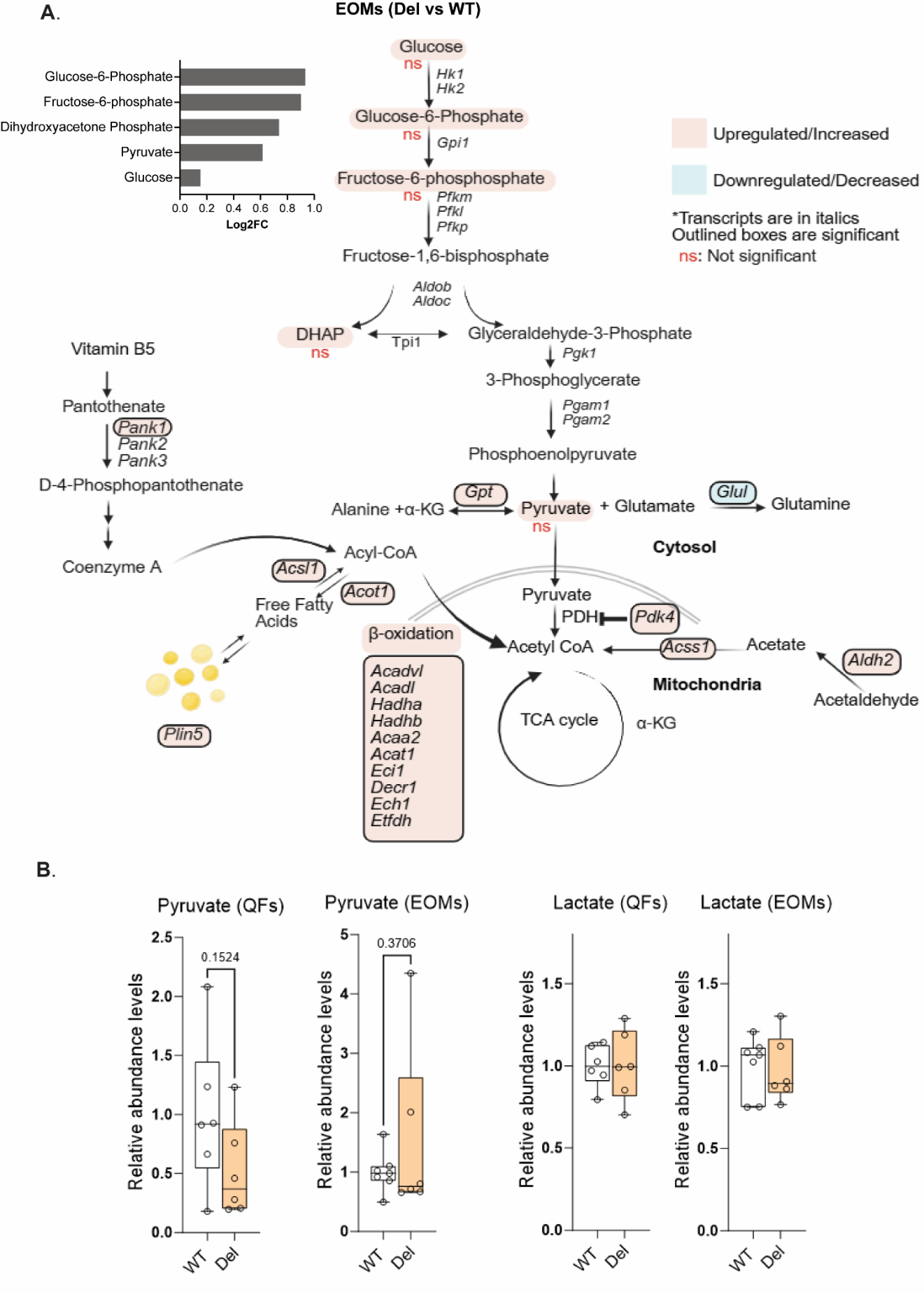
**

**Figure S5. Pyruvate and fatty acid metabolism are affected in EOMs with MM**

1. Schematic image shows changed transcripts and metabolites in Deletor EOMs. Red: increased and blue: decreased level. Transcripts are in italics. Outlined transcripts/metabolites are significantly changed (P < 0.05). Created with BioRender.com.
2. Pyruvate and Lactate relative abundance levels in QFs and EOMs.

Data information: In (B), p-value from t-test of Metabolomics dataset calculated by metaboanalyst; *P ≤ 0.05, **P ≤ 0.01, ***P ≤ 0.001, ****P ≤ 0.0001 (Student’s t-test)

| **Table S3: Primers used in this study** | | |
| --- | --- | --- |
| **Primer Sequences** | | |
| Analysis/genes |  | 5´-3´ |
| mtDNA deletions (long PCR) | Forward | GAGGTGATGTTTTTGGTAAACAGGCGGGGT |
|  | Reverse | GGTTCGTTTGTTCAACGAT TAAAGTCCTACGTG |
| mtDNA deletions (short PCR) | Forward | ACCCCGCCTGTTTACCAAAAACATCACCTC |
|  | Reverse | ACGTACCACTTTAATCGTTGAACAAACGAACC |
| MTRNR1 | Forward | AGGAGCCTGTTCTATAATCGATAAA |
|  | Reverse | GATGGCGGTATATAGGCTGAA |
| RBM15 | Forward | GGACAGTTTTCTTGGGCAAC |
|  | Reverse | AGTTTGGCCCTGTGAGACAT |
| FGF21 | Forward | CACCCAGGATTTGAATGACC |
|  | Reverse | GGGAGGATGGAACAGTGGT |
| Beta-Actin | Forward | ATGCTCCCCGGGCTGTAT |
|  | Reverse | CATAGGAGTCCTTCTGACCCATTC |
| DECR1 | Forward | GATCCGGGTCCTCAGAGGTTT |
|  | Reverse | ATCAGGTGGTAGCATAGGCTT |
| ETFDH | Forward | GTGCGACTAACCAAGCTGTC |
|  | Reverse | GGATGAACAGTGTAGTGAGTGG |

| **Table S4: Reagents and resources used in the study** | | | |
| --- | --- | --- | --- |
| REAGENT or RESOURCE | SOURCE | IDENTIFIER | Dilutions |

| Antibodies |
| --- |

| Rat-anti-LAMP1 | Santa Cruz | Cat#sc-19992 | 1:500 |
| --- | --- | --- | --- |
| Mouse-anti-p62 | Abcam | Cat#ab56416 | 1:400 |
| Anti-rat IgG Alexa ﬂuor 633 | Thermo Fisher Scientiﬁc | Cat#A-21094 | 1:500 |
| Anti-mouse IgG2a CF405S | Sigma | Cat#SAB4600476 | 1:400 |
| Rabbit-anti-phospho-S6 antibody | Cell Signaling Technology | Cat#2215 | 1:250 |
| Mouse-anti-MTCO1 | Abcam | Cat#ab14705 | 1:100 |
| Mouse-anti-SDHA | Abcam | Cat#ab14715 | 1:100 |
| Rabbit-anti-Phospho-Ser293-PDHA1 | Abcam | Cat#ab177461 | 1:250 |
| Rabbit-anti-MTHFD2 | Abcam | Cat# ab151447 | 1:300 |
| Anti-mouse IgG1 biotin | Abcam | Cat#ab97238 | 1:200 |
| Anti-mouse IgG2a Alexa Fluor™ 633 | Invitrogen | Cat#A21136 | 1:200 |
| Anti-rabbit IgG Alexa Fluor™ 568 | Invitrogen | Cat#A11011 | 1:200 |
| Streptavidin, Alexa Fluor™ 647 | Invitrogen | Cat#S21374 | 1:200 |
|  |  |  |  |

| Chemicals, peptides, and recombinant proteins |
| --- |

| Cytochrome c | Sigma Aldrich | Cat#C2506 |
| --- | --- | --- |
| Sodium succinate | Sigma Aldrich | Cat#S2378 |
| Catalase | Sigma Aldrich | Cat#C9322 |
| Nitro Blue Tetrazolium | Sigma Aldrich | Cat#N5514 |
| Paraformaldehyde | HUSLAB | Cat#E00323 |
| HEPES | Sigma Aldrich | Cat#H4034 |
| Sucrose | Thermo Fisher Scientiﬁc | Cat#10634932 |
| PBS | Corning | Cat#20-031-CV |
| Isopentane | Honeywell Research Chemicals | Cat#ZM32631 |
| O.C.T compound | Sakura | Cat#SA62550-01 |
| Bovine Serum Albumin | Thermo Fisher Scientiﬁc | Cat#BP9703100 |
| Triton-X | Sigma-Aldrich | Cat#X100-1L |
| Goat serum normal | Thermo Fisher Scientific | Cat#10000C |
| Tween 20 | Thermo Fisher Scientific | Cat# 10485733 |
| TBS tablets | Fisher Scientific | Cat#11300499 |
| Trizma base | Sigma-Aldrich | Cat# T1503-1KG |
| Trisodium citrate Dihydr Crystal | Sigma Aldrich | Cat#137042 |
| EDTA | Sigma-Aldrich | Cat#03609 |
| 3, 3 –diaminobenzidine (DAB) | Sigma Aldrich | Cat#D8001 |
| M.O.M (Mouse on Mouse) Blocking reagent | VectorLabs | Cat#MKB-2213-1 |
| Antibody Diluent, Background Reducing | Agilent, Dako | Cat#S3022 |
| Hoechst 33342 | Thermo Fisher Scientiﬁc | Cat#62249 |
| DRAQ5 | Abcam | Cat#ab108410 |
| Vectashield Antifade Mounting Medium | VectorLabs | Cat#H-1000 |
| Glutaraldehyde solution | Sigma Aldrich | Cat#G7651 |
| Proteinase K | Macherey-Nagel | Cat#740506 |
| Phusion High-Fidelity DNA Polymerase | Thermo Scientific | Cat#530L |
| SYBR Green Supermix | Bio-Rad | Cat#1725006CUST |
| Precellys Lysing Kit Ckmix | Bertin Technologies | Cat# P000918-LYSK0-A |
| TRIzol Reagent | Thermo Scientific | Cat#15596026 |
| Vectashield Antifade Mounting Medium | VectorLabs | Cat#H-1000 |
| SYBR Green Supermix | Bio-Rad | Cat#1725006CUST |
| 7-amino-actinomycin D | eBioscience | Cat#00-6993-50 |
|  |  |  |

| Critical commercial assays |
| --- |

| VECTASTAIN Elite ABC-Peroxidase Kit (rabbit IgG) | VectorLabs | Cat#PK-6101 |
| --- | --- | --- |
| RNeasy Mini Kit | Qiagen | Cat#74104 |
| Maxima First Strand cDNA Synthesis Kit for RT-qPCR | Thermo Scientific | Cat#K1671 |
|  |  |  |

| Deposited data |
| --- |

| Bulk-RNAseq Dataset | European nucleotide archive (ENA) | Accession no. PRJEB73353 |
| --- | --- | --- |
| Metabolomics Dataset | Metabolomics workbench | Study ID ST003145 |

|  |  |  |
| --- | --- | --- |
| Experimental models: Organisms/strains | | |

| Deletor (Tg/ACTB/twnk-p.353-365-dup/BL6) | Suomalainen Lab^1^ | N/A |
| --- | --- | --- |
| *mito-QC* (Tg/mCherry-GFP-Fis1^101-152^/BL6) | McWilliams Lab^2^ | N/A |
|  |  |  |

| Software and algorithms |
| --- |

| Ilastik 1.4.0 software | Berg et al.^3^ | https://www.ilastik.org/ |
| --- | --- | --- |
| CellProfiler 4.2.5 software | McQuin et al.^4^ | https://cellprofiler.org/ |
| DESeq2 | Love et al. ^5^ | https://bioconductor.org/packages/release/bioc/html/DESeq2.html |
| R environment 4.2.0 | R core team | https://www.r-project.org/ |
| Python | Scanpy | https://scanpy.readthedocs.io/en/stable/ |
| Metaboanalyst | Pang et al.^6^ | https://www.metaboanalyst.ca/ |

| Prism version 9.3.0 | Graphpad Software | https://www.graphpad.com/ |
| --- | --- | --- |
| Other | | |
| Mouse diet | Altromin Spezialfutter | C1000 |

**References**

1. Tyynismaa H, Mjosund KP, Wanrooij S, et al. Mutant mitochondrial helicase Twinkle causes multiple mtDNA deletions and a late-onset mitochondrial disease in mice. Proc. Natl. Acad. Sci. U. S. A. 2005;102(49):17687–17692.

2. McWilliams TG, Prescott AR, Allen GFG, et al. Mito-QC illuminates mitophagy and mitochondrial architecture in vivo. J. Cell Biol. 2016;214(3):333–345.

3. Berg S, Kutra D, Kroeger T, et al. Ilastik: Interactive Machine Learning for (Bio)Image Analysis. Nat. Methods 2019;16(12):1226–1232.

4. McQuin C, Goodman A, Chernyshev V, et al. CellProfiler 3.0: Next-generation image processing for biology. PLOS Biol. 2018;16(7):e2005970.

5. Love MI, Huber W, Anders S. Moderated estimation of fold change and dispersion for RNA-seq data with DESeq2. Genome Biol. 2014;15(12):1–21.

6. Pang Z, Chong J, Zhou G, et al. MetaboAnalyst 5.0: narrowing the gap between raw spectra and functional insights. Nucleic Acids Res. 2021;49(W1):W388–W396.
